# Geographical Disparities and Socioeconomic Determinants of Untreated Dental Caries in Permanent Teeth Across Asia, 1990–2019: A Retrospective Population‐Based Burden Analysis

**DOI:** 10.1155/ijod/5549710

**Published:** 2026-01-28

**Authors:** Huang Cui, Li Minsi, Ma Ri, Huang Wanping, Pang Fanghe, Qin Xiaofeng

**Affiliations:** ^1^ College and Hospital of Stomatology, Guangxi Medical University, 10 Shuangyong Road, Qingxiu District, Nanning, 530021, China, gxmu.edu.cn; ^2^ Guangxi Clinical Research Center for Craniofacial Deformity, College of Stomatology, Hospital of Stomatology, Guangxi Medical University, Nanning, China, gxmu.edu.cn; ^3^ Medicine and Technology College of ZUNYI Medical University, Zunyi, China; ^4^ Guangxi Key Laboratory of Oral and Maxillofacial Rehabilitation and Reconstruction, College of Stomatology, Hospital of Stomatology, Guangxi Medical University, Nanning, China, gxmu.edu.cn

**Keywords:** caries, epidemiology, prevention, public health

## Abstract

**Objectives:**

We aimed to analyze the trends of untreated caries in permanent teeth in Asia over the past 30 years, identify high‐risk groups and areas of high prevalence, and explore strategies to mitigate this disease burden.

**Methods:**

Data were extracted from the Global Burden of Disease (GBD) Database. The incidence, prevalence, years lived with disability (YLDs), and age‐standardized incidence rate (ASIR), age‐standardized prevalence rate (ASPR), and age‐standardized YLD rate (ASYR) were analyzed. Spatial and temporal distributions of untreated caries in permanent teeth were also determined.

**Results:**

For Asia, the crude incidence, prevalence, and YLDs showed increasing trends; ASIR remained constant; and ASPR and ASYR demonstrated decreasing trends. In 2019, the incidence and prevalence of untreated caries in permanent teeth peaked in the 20–24‐year age group. The spatial “hot spots” for ASIR were South and East Asia, whereas the “cold spots” were West and Central Asia. The hot spots for ASPR and ASYR were in West Asia, whereas the cold spots were in East Asia.

**Conclusions:**

Untreated caries in permanent teeth demonstrated significant geographical and demographic differences in Asia, revealing a relevant public health challenge. Intervention strategies based on dynamic changes are required to reduce the burden of dental caries in this region.

## 1. Introduction

Dental caries in permanent teeth is the most prevalent noncommunicable disease globally, imposing significant health and socioeconomic challenges. In 2019, untreated caries affected ~3.088 billion individuals worldwide—nearly 40% of the global population. Asia accounted for >60% of this burden, equating to 2 million years lived with disability (YLDs) [1–3]. Despite rapid economic growth in many Asian countries, oral health improvements remain uneven, as disparities in healthcare access, dietary patterns, and socioeconomic development persist across subregions. These inequities are further compounded by the rising consumption of sugar‐sweetened beverages and processed foods [4].

Chronic pain, tooth loss, and functional impairments diminish the quality of life and limit educational and economic opportunities, particularly among low‐income populations who face prohibitive out‐of‐pocket healthcare costs [5, 6]. Additionally, financial strains on families and healthcare systems underscore the urgency of addressing this preventable disease. While global guidelines emphasize sugar reduction and preventive care, policy implementation in Asia remains fragmented, leaving vulnerable groups disproportionately exposed to risk [7].

Epidemiological studies have largely focused on national‐level trends, neglecting critical subregional variations and spatial clustering of disease burden. For example, high incidence rates in South and East Asia contrast sharply with elevated prevalence in West Asia [8]. Such regional nuances are essential for designing targeted interventions. Furthermore, few studies have integrated socioeconomic determinants—including the sociodemographic index (SDI), dentist density, and smoking prevalence—with spatiotemporal analyses, limiting actionable insights for policymakers [9].

To address these gaps, this study comprehensively analyzed the burden of untreated caries across 47 Asian countries from 1990 to 2019. Using spatial epidemiology and multivariable regression, we quantified temporal trends in incidence, prevalence, and YLDs at regional and subregional levels; identified geographical hotspots and key sociodemographic drivers; and proposed context‐specific strategies to mitigate inequities. This study not only bridges critical knowledge gaps but also provides an evidence base for prioritizing prevention, expanding treatment access, and addressing systemic barriers to oral health equity in Asia.

## 2. Methods

### 2.1. Data Sources and Indicators

Data on the age‐standardized incidence rate (ASIR), age‐standardized prevalence rate (ASPR), and age‐standardized YLDs rate (ASYR) for untreated dental caries in permanent teeth across 47 Asian countries from 1990 to 2019 were obtained from the Global Burden of Disease Study 2019 (GBD 2019). All datasets were downloaded from the Institute for Health Metrics and Evaluation’s official website (https://vizhub.healthdata.org/gbd-results/). The query parameters were defined as follows: measure—incidence, prevalence, and YLD; cause—untreated dental caries; locations—47 Asian countries; years—1990–2019; age—all ages; sex: both; and metric—rate per 100,000 population.

Additional country‐level covariates included the SDI, dentist density per 10,000 population (DD10k), daily estimated intake of sugar‐sweetened beverages among adults aged ≥25 years (DEISSB25), and age‐standardized smoking prevalence among individuals aged ≥15 years (A‐SSP15). Age‐standardized rates were calculated using the GBD 2019 global standard population. These covariates were extracted from the same GBD 2019 database (https://ghdx.healthdata.org/gbd-2019) following consistent standardization procedures.

As oral diseases seldom result in mortality, this study focused on YLDs rather than disability‐adjusted life years. The incidence‐to‐prevalence ratio (I/P ratio) was used as a proxy indicator for treatment coverage. The SDI was calculated as the geometric mean of the rescaled values for income per capita, educational attainment, and total fertility rate, ranging from 0 (lowest development) to 1 (highest development) [7, 10].

### 2.2. Definition of Untreated Dental Caries

This study considered only caries in permanent teeth. According to the World Health Organization (WHO) and GBD criteria, untreated dental caries was defined as a tooth with a visible dentinal cavity (coronal) or a soft/leathery root lesion upon probing. In the GBD classification, this corresponds to ICD‐9 code 521.0 and ICD‐10 codes K02.3–K02.9 [2, 7].

### 2.3. Spatial Autocorrelation Analysis

We examined spatial patterns of disease burden using both global and local spatial autocorrelation analyses. Global Moran’s I was used to evaluate overall spatial clustering, with values ranging from −1 (perfect dispersion) to + 1 (perfect clustering). Statistical significance was determined using Z‐scores and *p*‐values [11]. To identify local clusters, Getis–Ord Gi ^∗^ statistics were applied to detect “hot” and “cold” spots of ASIR, ASPR, and ASYR. A Z‐score >1.96 (*p* < 0.05) indicated significant local clustering. Analyses were performed using ArcGIS 10.3 (ESRI, Redlands, CA, USA) [12, 13].

### 2.4. Spatial Regression Analysis

To explore spatial associations between disease burden and socioeconomic factors, we applied both ordinary least squares (OLSs) and geographically weighted regression (GWR) models, controlling for spatial dependance [11, 14]. OLS models were used to assess global relationships among ASIR, ASPR, ASYR, and the four independent variables (SDI, DD10k, DEISSB25, and A‐SSP15). Model performance was evaluated using adjusted *R* ^2^, Akaike Information Criterion, Bayesian Information Criterion, and residual spatial autocorrelation.

Subsequently, we used GWR to identify spatially varying associations, estimating localized coefficients for each independent variable across geographic units using GeoDa (v1.12.0), QGIS (v2.14), and RStudio (v1.1.463). Choropleth maps were created to visualize the spatial distributions of ASIR, ASPR, ASYR, and the GWR coefficients.

## 3. Results

### 3.1. General Trends in Untreated Caries in Permanent Teeth in Asia

The age‐standardized and crude incidence, prevalence, and YLD rates of untreated caries in permanent teeth in Asia during 1990–2019 showed an upward trend in incidence, a stable trend in ASIR, and downward trends in ASPR and ASYR (Figure 1).

Figure 1Crude and age‐standardized incidence (a) and prevalence (b) of untreated dental caries in permanent teeth in Asia, 1990–2019, and temporal trends in years lived with disability (YLDs) (c).

**Figure:**
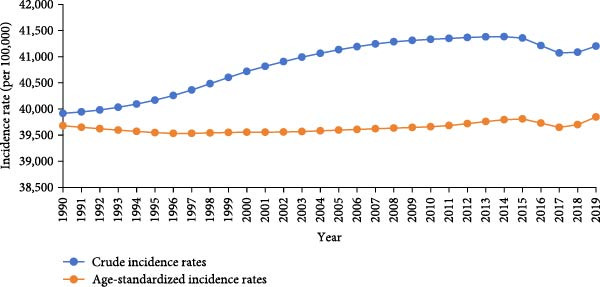
(a)

**Figure:**
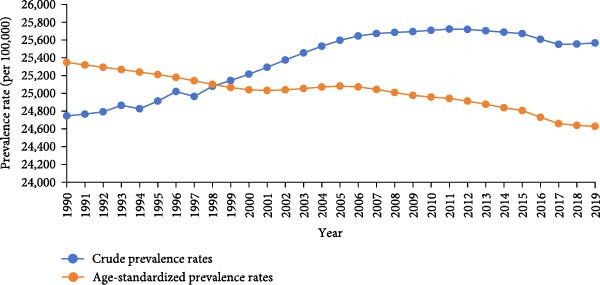
(b)

**Figure:**
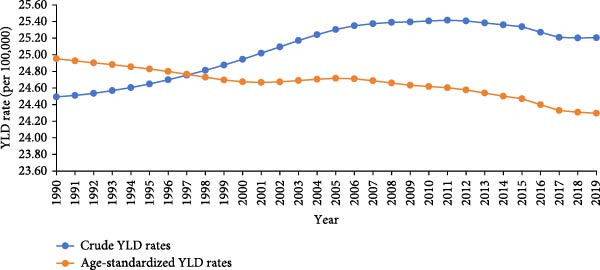
(c)

**Figure 2:**
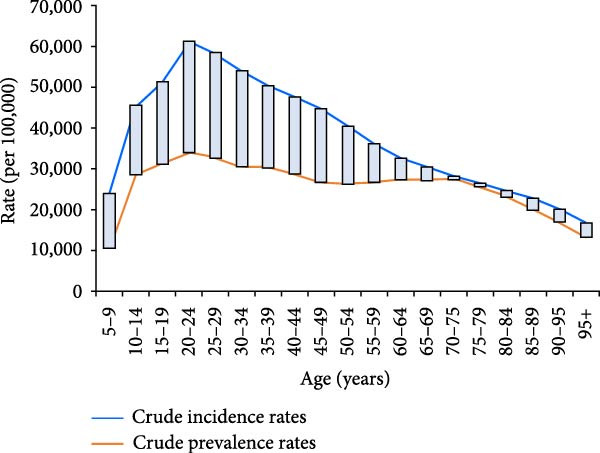
Age‐specific incidence and prevalence of untreated dental caries in permanent teeth in the Asian population, 2019.

The age distributions are shown in Figure 2. The lowest incidence rate was observed in the ≥95‐year age group, rapidly increasing after 5 years of age, and peaking in the 20–24‐year age group (incidence rate, 58305.92/100,000; prevalence rate, 34612.17/100,000). The largest and smallest differences between the prevalence and incidence were observed in the 20–24‐ and 70–75‐year groups, respectively.

### 3.2. Global Spatial Autocorrelation

The global spatial autocorrelation results of the ASIR, ASPR, and ASYR are presented in Table 1. All *Z*‐scores were >0, and all *p*‐values were <0.05, indicating a spatial correlation among the incidence, prevalence, and YLD of untreated permanent dental caries in Asia. Moreover, Moran’s *I* values were >0, indicating a positive spatial correlation. The Moran’s *I* values for ASPR and ASYR were higher than those for ASIR, indicating that the spatially positive correlation between ASPR and ASYR was stronger than that between ASIR and ASYR.

**Table 1:** Global spatial autocorrelation of age‐standardized incidence rate (ASIR), age‐standardized prevalence rate (ASPR), and age‐standardized years lived with disability rate (ASYR) of untreated caries of permanent teeth in Asia, 1990–2019.

Year	Moran’s *I*	*Z* score	*p*‐Value
ASIR			
1990	0.42	3.55	0.001
1995	0.57	4.77	0.001
2000	0.37	3.18	0.004
2005	0.43	3.79	0.001
2010	0.31	2.82	0.001
2015	0.28	2.53	0.011
2019	0.29	2.60	0.008
ASPR			
1990	0.66	5.68	0.001
1995	0.63	5.42	0.001
2000	0.60	5.15	0.001
2005	0.59	5.01	0.001
2010	0.63	5.42	0.001
2015	0.66	5.68	0.001
2019	0.68	5.85	0.001
ASYR			
1990	0.68	5.81	0.001
1995	0.64	5.53	0.001
2000	0.62	5.27	0.001
2005	0.59	5.09	0.001
2010	0.64	5.49	0.001
2015	0.67	5.73	0.001
2019	0.68	5.89	0.001

### 3.3. Local Spatial Autocorrelation

#### 3.3.1. ASIR

The “hot” and “cold” spots in the ASIR are shown in Figure 3a. Hot spots were mainly concentrated in South and East Asia, and the range gradually narrowed over time. Since 2010, hotspots have stabilized in South Asian countries, including India, Myanmar, Vietnam, and Cambodia. Cold spots were mainly concentrated in West and Central Asia and gradually shifted from the West to Central Asia. Recently, the scope of cold spots has gradually narrowed to Central Asian countries, including Uzbekistan, Tajikistan, and Kyrgyzstan.

Figure 3Spatial clusters (hot and cold spots) of age‐standardized incidence (ASIR) (a), prevalence (ASPR) (b), and years lived with disability rates (ASYR) (c) of untreated dental caries in permanent teeth in Asia, 1990–2019. Classification method: Getis–Ord Gi ^∗^ statistic, significance levels at *p* < 0.05.

**Figure:**
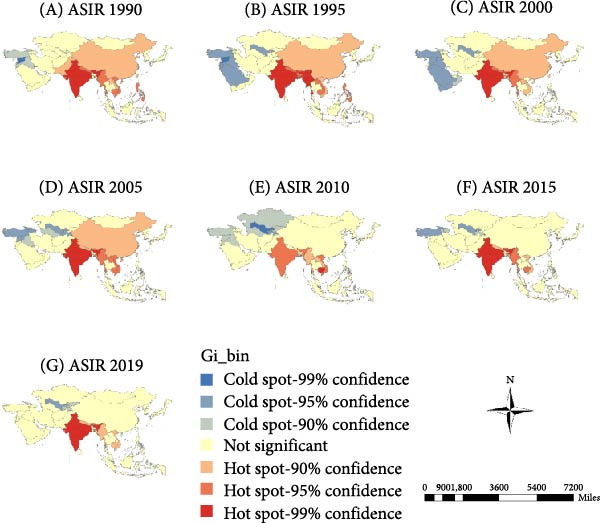
(a)

**Figure:**
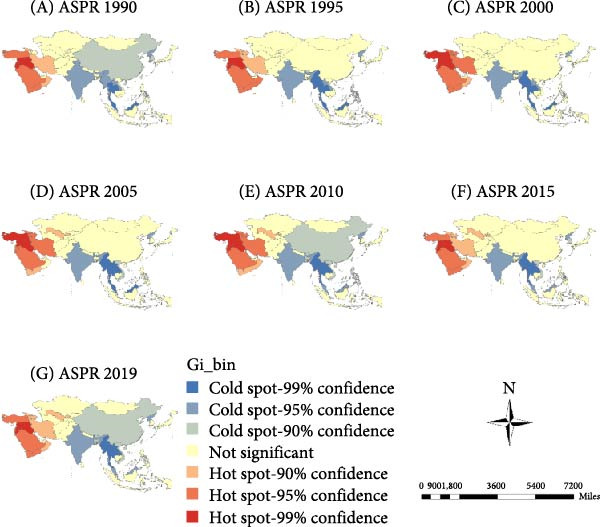
(b)

**Figure:**
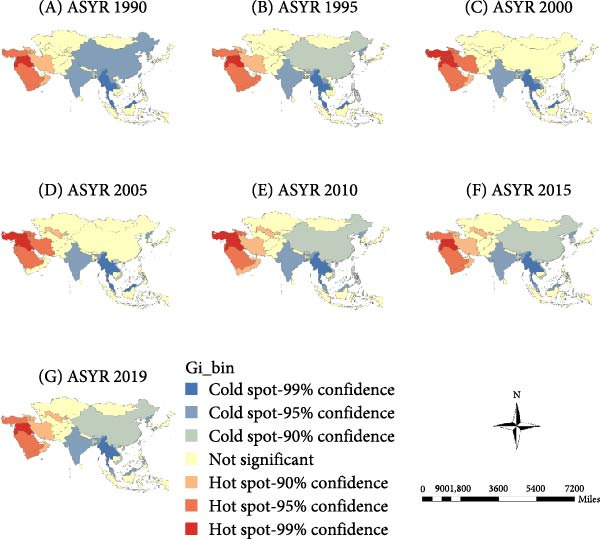
(c)

#### 3.3.2. ASPR

The ASPR showed hot spots concentrated in West Asian countries, including Turkey, Syria, Iraq, and Saudi Arabia, with Uzbekistan in Central Asia emerging as a new hot spot since 2005. Cold spots were mainly concentrated in China in East Asia, and India, Myanmar, Thailand, and Malaysia in South Asia (Figure 3b).

#### 3.3.3. ASYR

ASYR hot spots were mainly concentrated in West Asian countries, including Turkey, Syria, Iraq, and Saudi Arabia, and in Uzbekistan in Central Asia in 2005. Cold spots were mainly concentrated in China in East Asia, and India, Myanmar, Thailand, and Malaysia in South Asia (Figure 3c).

### 3.4. Factors Influencing Untreated Caries in Permanent Teeth Based on OLS

The *R* ^2^ values of the OLS regression models for the factors affecting the ASIR of untreated caries in permanent teeth in Asia in 1990, 2000, 2010, and 2019 were 0.15, 0.13, 0.12, and 0.12, respectively, whereas those for ASPR and ASYR were 0.23, 0.18, 0.17, and 0.15 and 0.22, 0.17, 0.17, and 0.14, respectively. Higher DEISSB25 was associated with higher ASPR of untreated caries in permanent teeth in 2000, 2010, and 2019 (*p* < 0.05). In contrast, higher SDI was associated with lower ASPR of untreated caries in permanent teeth in 1990, 2000, and 2010 (*p* < 0.05). Higher DEISSB25 was associated with higher ASYR of untreated caries in permanent teeth in 2000, 2010, and 2019, whereas higher SDI was associated with lower ASYR in 1990, 2000, and 2010 (all *p* < 0.05) (Table 2).

**Table 2:** Ordinary least squares regression of age‐standardized incidence rate (ASIR), age‐standardized prevalence rate (ASPR), and age‐standardized years lived with disability rate (ASYR) of untreated caries of permanent teeth in Asia, 1990–2019.

Factor	1990			2000			2010			2019		
	*β*	*t*	*p*	*β*	*t*	*p*	*β*	*t*	*p*	*β*	*t*	*p*
ASIR												
*X*_1_	2767.57	1.46	0.15	3817.84	1.70	0.10	3319.60	1.23	0.23	1641.33	0.74	0.46
*X*_2_	2.55	0.61	0.55	1.61	0.33	0.74	5.83	1.22	0.23	3.21	0.82	0.42
*X*_3_	−1837.14	−1.35	0.18	−1985.00	−1.26	0.21	−1916.37	−1.02	0.31	−1927.87	−1.11	0.27
*X*_4_	−94.66	−0.71	0.48	−18.97	−0.17	0.87	−105.53	−1.10	0.28	−64.64	−0.91	0.37
ASPR												
*X*_1_	−11437.65	−1.73	0.09	−7139.63	−0.91	0.37	30.64	0.00	1.00	3340.18	0.40	0.69
*X*_2_	25.96	1.78	0.08	36.13	2.13	0.04 ^∗^	37.28	2.49	0.02 ^∗^	35.09	2.41	0.02 ^∗^
*X*_3_	−10586.23	−2.23	0.03 ^∗^	−12548.91	−2.27	0.03 ^∗^	−13714.79	−2.34	0.02 ^∗^	−12942.05	−1.99	0.05
*X*_4_	681.69	1.47	0.15	324.90	0.81	0.42	245.23	0.81	0.42	154.78	0.58	0.56
ASYR												
*X*_*1*_	−11.06	−1.70	0.10	−6.89	−0.89	0.38	0.27	0.03	0.97	3.71	0.46	0.65
*X*_*2*_	0.03	1.77	0.08	0.04	2.08	0.04 ^∗^	0.04	2.46	0.02 ^∗^	0.03	2.41	0.02 ^∗^
*X*_*3*_	−9.73	−2.09	0.04 ^∗^	−11.57	−2.12	0.04 ^∗^	−12.98	−2.25	0.03 ^∗^	−12.28	−1.93	0.06
*X*_*4*_	0.65	1.42	0.16	0.30	0.77	0.45	0.24	0.80	0.43	0.15	0.57	0.57

*Note: X*_1_ = Age‐standardized smoking prevalence among individuals aged ≥15 years, *X*_2_ = daily individual intake of sugar‐sweetened beverages estimates among individuals aged ≥25 years, *X*_3_ = sociodemographic index (SDI) values, *X*_4_ = dentist density per 10,000 population;  ^∗^ *p* < 0.05.

### 3.5. Factors Affecting Untreated Caries in Permanent Teeth Based on GWR

The *R* ^2^ values of the GWR for ASIR of the factors affecting untreated caries in permanent teeth in 1990, 2000, 2010, and 2019 were 0.24, 0.19, 0.20, and 0.23, respectively, whereas those for ASPR and ASYR were 0.30, 0.25, 0.26, and 0.28 and 0.29, 0.24, 0.26, and 0.28, respectively, with an improved goodness‐of‐fit over OLS in the same year.

A‐SSP15 was positively associated with the ASIR in all years. GWR coefficients were higher in West and Central Asian countries in 1990 and 2000, and in East and Southeast Asian countries in 2010 and 2019, while consistently lower coefficients were observed in South Asian countries (Figure 4a A–D).

Figure 4Spatial distributions of geographically weighted regression (GWR) coefficients of factors influencing age‐standardized burden indicators (a: ASIR, b: ASPR, and c: ASYR) of untreated caries in permanent teeth in Asia, 1990–2019. (A–D) Age‐standardized smoking prevalence among individuals ≥15 years (1990, 2000, 2010, and 2019); (E–H) Daily individual intake of sugar‐sweetened beverages among individuals ≥25 years; (I–L) Sociodemographic Index (SDI) values; (M–P) Dentist density per 10,000 population. Classification method: GWR coefficients classified based on quantiles; warm colors represent stronger positive associations, while cool colors show negative associations.

**Figure:**
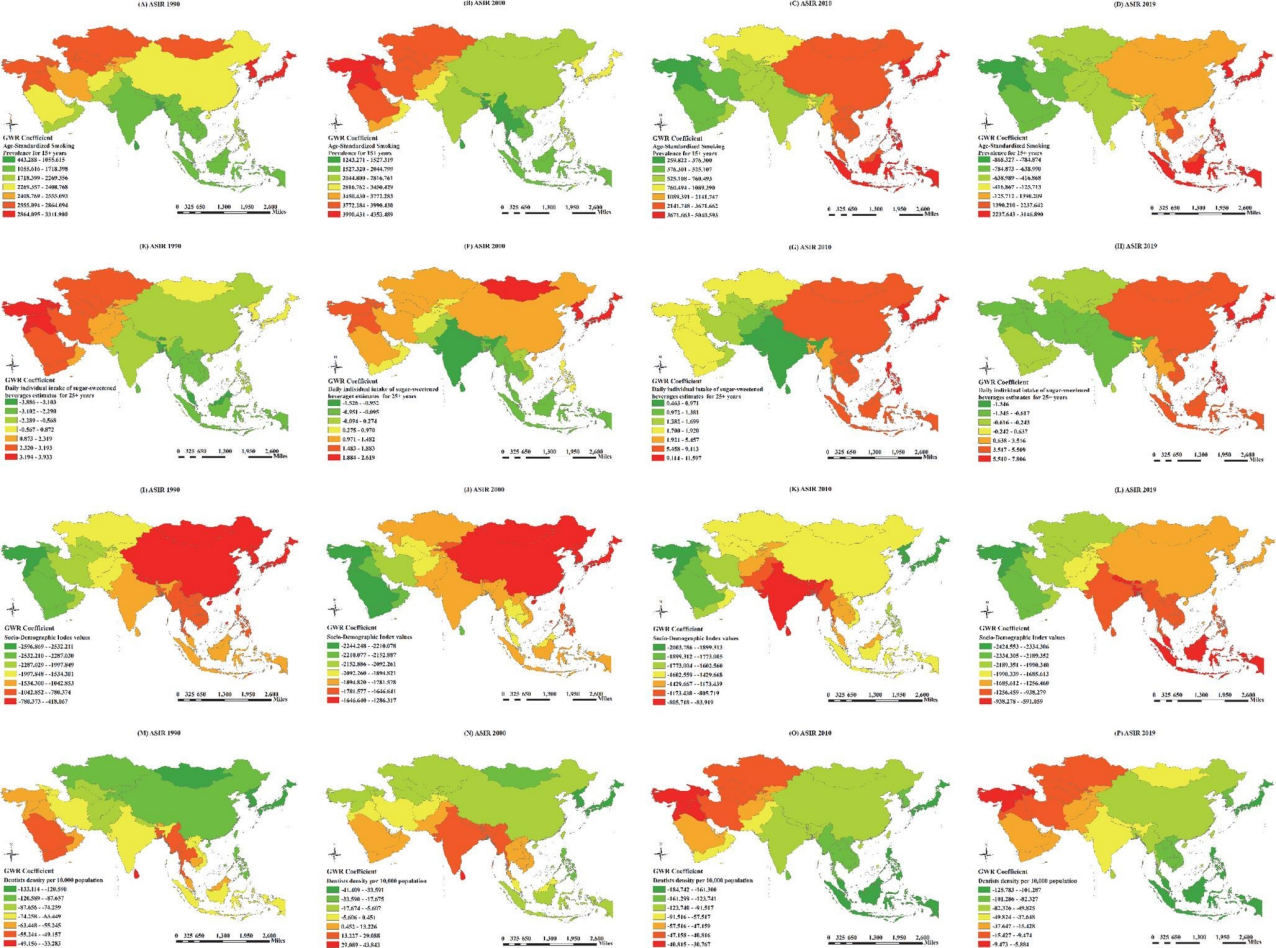
(a)

**Figure:**
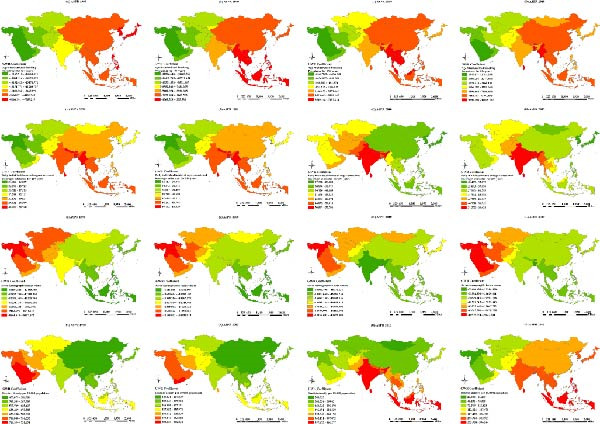
(b)

**Figure:**
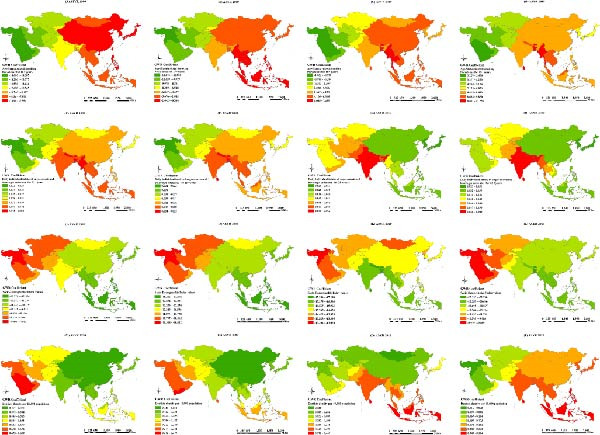
(c)

DEISSB25 was positively associated with the ASIR. GWR coefficients were higher in West, Central, and East Asian countries in 1990 and 2000, and in East and Southeast Asian countries in 2010 and 2019, while consistently lower coefficients were noted in South Asian countries (Figure 4a E–H).

The SDI was negatively associated with the ASIR in all years, with higher GWR coefficients observed for all years in West Asian countries (Figure 4a I–L). DD10k was predominantly negatively associated with the ASIR, with higher GWR coefficients in East Asian countries in 1990 and 2000 and Southeast Asian countries in 2010 and 2019 (Figure 4a M–P).

A‐SSP15 was predominantly negatively associated with the ASPR and ASYR, with higher GWR coefficients in West Asian countries and consistently lower values in East and Southeast Asian countries (Figure 4b, c A–D). DEISSB25 was positively associated with the ASPR and ASYR in all years, with higher GWR coefficients in South and Southeast Asian countries in 1990 and 2000, and South Asian countries in 2010 and 2019 (Figure 4b, c E–H). The SDI was negatively associated with the ASPR and ASYR, with consistently higher GWR coefficients in East and Southeast Asian countries and consistently lower values in West and Central Asian countries (Figure 4b, c I–L). DD10k was positively associated with the ASPR and ASYR in all years, with higher GWR coefficients in West Asian countries in 1990 and 2000, and South and Southeast Asian countries in 2010 and 2019 (Figure 4b, c M–P).

## 4. Discussion

This study is the first to comprehensively reveal the long‐term spatial patterns and socioeconomic determinants of the burden of untreated dental caries in permanent teeth across 47 Asian countries during 1990–2019. The findings address key knowledge gaps regarding regional disparities and the role of social determinants, providing an evidence‐based foundation for precise public health interventions and oral health planning.

### 4.1. Temporal Trends Highlight High‐Risk Age Groups

Although the ASIR of untreated dental caries in permanent teeth remained stable over three decades, the crude incidence, prevalence, and YLD rates increased, largely driven by population aging. The burden peaked in the 20–24 age group, with the largest difference between incidence and prevalence, suggesting relatively high treatment accessibility among younger adults. In contrast, the 70–74 age group showed high prevalence but low incidence and minimal difference between the two, indicating inadequate treatment coverage and a growing burden among older adults.

This finding highlights the need for a life‐course approach to oral health, emphasizing both early prevention and equitable access to care across all age groups. For older adults, factors, including multimorbidity, reduced manual dexterity, and financial barriers, contribute to poor oral health outcomes, underscoring the need for targeted public policies and service delivery innovations to strengthen oral health support in this population [5, 15]. Simultaneously, for younger adults, particularly those aged 20–24 years who showed rising incidence rates, effective sugar control strategies are urgently needed. Public health efforts, including sugar taxation, regulation of sugary food marketing, and health education campaigns, should be implemented to mitigate dietary risk factors and curb the early onset of dental caries [3].

### 4.2. Spatial Clustering Reflects Inequities in Oral Health Across Asia

Spatial autocorrelation analysis revealed nonrandom geographic clustering of ASIR, ASPR, and ASYR. ASIR hot spots were mainly concentrated in South and East Asia, while cold spots gradually shifted from West to Central Asia. For ASPR and ASYR, hot spots were identified in West Asia (e.g., Turkey, Iraq, and Saudi Arabia), whereas cold spots were concentrated in East and South Asia (e.g., China and India). These opposing spatial patterns suggest a discrepancy between caries onset and disease management across regions. Notably, the high ASIR yet low ASPR and ASYR observed in East and South Asia indicate a higher incidence of new cases but more effective control or timely treatment, specifically an “acute‐high” but “well‐managed” disease burden. Conversely, regions with low ASIR but high ASPR and ASYR, such as West Asia, likely experience delayed or inadequate treatment, leading to a “chronic‐persistent” disease burden. This contrast may reflect differences in oral health infrastructure, socioeconomic development, healthcare accessibility, and public health priorities.

Such geographic disparities are frequently rooted in structural inequalities. For example, in 2019, the United Arab Emirates had one of the highest SDI scores in Asia, whereas countries, including Afghanistan, ranked among the lowest [8, 16]. Inadequate integration of oral health into primary care systems and insufficient universal coverage in parts of Asia have also contributed to disparities in disease management [17]. Political instability, limited dental workforce, and weak health systems in some countries may hinder access to timely and effective oral healthcare, resulting in the accumulation of untreated cases [18]. These findings underscore the need for targeted policy interventions and international cooperation to improve oral health equity, particularly in underserved and politically unstable regions.

Further epidemiological and health systems research is warranted to identify the specific drivers of these spatial inequalities and inform evidence‐based strategies for caries prevention, early detection, and treatment across Asia.

### 4.3. Spatial Heterogeneity in the Effects of Socioeconomic and Commercial Determinants

GWR revealed significant spatial heterogeneity in the association between the SDI and caries burden. A lower SDI was observed alongside higher ASIR in West Asia and higher ASPR and ASYR in East and Southeast Asia. These findings underscore the vulnerability of populations in low‐resource settings, where weak public health systems, limited preventive services, and high out‐of‐pocket costs are frequently present alongside restricted access to timely dental care [9].

Moreover, higher DEISSB25 was consistently associated with higher ASIR, ASPR, and ASYR. This aligns with the well‐established link between sugar consumption and dental caries, which exhibits a clear dose–response relationship [19]. However, sugar intake below the WHO’s recommended limit (<10% of total energy) remains associated with significantly increased caries risk, suggesting that no safe threshold exists for sugar consumption in caries prevention [20].

Higher A‐SSP15 was associated with higher ASIR but lower ASPR and ASYR. Smoking is linked to an increased caries risk, potentially through mechanisms, including altered salivary flow and the promotion of cariogenic microbial environments [21, 22]. Smoking is also correlated with an increased risk of tooth loss due to severe periodontal disease [22, 23], which may paradoxically reduce the number of teeth at risk for caries in smokers, particularly among older adults. Given the persistently high smoking rates in several Southeast Asian countries [24], implementing comprehensive tobacco control strategies—including taxation, public smoking bans, and educational campaigns—is an urgent public health priority.

### 4.4. Structural Challenges in the Current Oral Healthcare System

Higher DD10k was associated with lower ASIR but higher ASPR and ASYR, suggesting a potential mismatch between service availability and oral health outcomes [25]. This paradox may reflect the treatment‐centric nature of current oral health systems, which operate under fee‐for‐service models that tend to emphasize restorative rather than preventive care [5, 26]. Therefore, while the incidence of new caries may decline in contexts with increased treatment availability, the prevalence of untreated caries remains high in settings with inequitable access and limited emphasis on disease prevention.

Improving outcomes and reducing inequities requires a shift towards value‐based care models that prioritize health outcomes over service volume and encourage upstream prevention rather than downstream intervention [27].

### 4.5. Broader Contextual Factors: Fluoride Exposure, Oral Health Policy, and Health Expenditure

Beyond socioeconomic status and behavioral determinants, other contextual factors—including fluoride exposure, national oral health policies, and healthcare expenditure—also influence the burden of untreated dental caries across Asia.

Fluoride exposure is one of the most cost‐effective public health measures for caries prevention [28]. However, its implementation varies across regions. Although water fluoridation exists in Singapore and parts of Malaysia, it is rare in most low and middle‐income countries due to infrastructural and political barriers [29]. Conversely, endemic fluorosis in parts of India, China, and Pakistan reduces public acceptance of fluoridation. The lack of standardized fluoride delivery may partly explain spatial heterogeneity in caries burden. Affordable interventions, including subsidized fluoridated toothpaste and school‐based topical fluoride programs, could improve prevention [30].

Integrating oral health policy into broader healthcare systems also plays a pivotal role. Countries that have embedded oral health into national universal health coverage (UHC) frameworks, such as Japan and South Korea, exhibit lower rates of untreated dental caries than those where dental care remains largely privatized [31]. In contrast, many low‐SDI regions exclude dental services from essential health benefit packages, leading to high out‐of‐pocket expenditures and delayed care‐seeking behavior [17]. According to the WHO 2023 Global Oral Health Report, fewer than one‐third of Asian countries possess national oral health policies aligned with UHC objectives, revealing a significant gap in implementation [32].

Healthcare expenditure shapes system capacity and access. In Asia, health expenditure as a proportion of gross domestic product (GDP) closely aligns with workforce and service use. High‐income regions, including Japan and Singapore, invest >9% of GDP, sustaining strong preventive and restorative systems. In contrast, many South and West Asian countries spend <4%, resulting in workforce shortages and limited coverage. Inadequate public financing remains a major barrier to equity in the WHO Southeast Asia region. The elevated ASPR and ASYR in West Asia likely reflect chronic underinvestment in primary oral care. Expanding public funding and performance‐based preventive programs could improve both efficiency and equity [33, 34].

Overall, these contextual factors highlight that reducing untreated dental caries in Asia requires addressing individual risk behaviors and advancing systemic reforms. Integrating fluoride‐based prevention, universal oral health coverage, and sustainable financing into regional health strategies represents an essential pathway toward oral health equity.

### 4.6. Region‐Specific Policy Implications

Based on the distinct spatial patterns and drivers of untreated caries identified in our analysis, policy responses should be subregion‐specific:

In East Asia, the pattern of high ASIR but low ASPR and ASYR suggests effective case management but insufficient primary prevention. Therefore, policies should prioritize curbing new cases through community‐wide fluoride programs and the integration of sugar control measures, aligning with the region’s significant association between sugar intake (DEISSB25) and ASIR. In South Asia, a persistent hotspot for ASIR, interventions should directly address the region’s high incidence. This requires integrating oral health into primary care to improve access, alongside fiscal and regulatory policies to reduce sugar consumption, a key driver identified by our GWR models. In West Asia, the high ASPR and ASYR hotspots indicate a critical backlog of untreated disease and health system failure in providing timely care. Policy should focus on expanding restorative service coverage through public insurance and investing in outreach programs to manage the accumulated burden. In Central Asia, the emergence of ASPR hotspots highlights the need for foundational health system strengthening. Priorities include addressing workforce shortages through regional cooperation and promoting oral health literacy to prevent a future surge in incidence.

### 4.7. Limitations

This study used estimates from the GBD 2019 database, which, despite robust modeling techniques, may differ from national data sources due to methodological differences. Additionally, our analysis was limited to data through 2019 because the DD10k, A‐SSP15, and DEISSB25 datasets have not yet been updated in subsequent GBD releases, such as GBD 2021. Therefore, we used the 2019 dataset to ensure data consistency and methodological reliability. Future studies should incorporate the latest data releases once these key input sources are updated, to enable more accurate comparisons and trend validations. Despite these limitations, our study offers valuable insights into the spatial and temporal dynamics of untreated caries in permanent teeth across Asia and presents actionable recommendations for reducing oral health inequalities.

## 5. Conclusions

The crude rate of untreated caries in permanent teeth increased between 1990 and 2019 in Asia. The largest and smallest differences between prevalence and incidence were observed in the 20–24‐ and 70–74‐year age groups, respectively, with significant spatial inequalities between countries. South and East Asia should pay more attention to caries prevention, whereas West Asia should invest more in treatment. Caries risk was negatively and positively correlated with SDI and sugar consumption, respectively; it also showed strong associations with dentist density and smoking prevalence. These findings suggest that policymakers should give more attention to older people and adopt more effective methods to address sugar consumption and smoking.

## Ethics Statement

The authors have nothing to report.

## Consent

The authors have nothing to report.

## Conflicts of Interest

The authors declare no conflicts of interest.

## Author Contributions

Huang Cui, Li Minsi, and Ma Ri contributed equally and share first authorship.

## Funding

The authors declare that financial support was received for the research, authorship, and/or publication of this article. This work was supported by the National Clinical Key Specialty Construction Project (No. CZ000037).

## Data Availability

The datasets used in this study are available from the Global Burden of Disease (GBD) database at https://ghdx.healthdata.org/gbd-results-tool.
